# Metabolic Host–Microbiota Interactions in Autophagy and the Pathogenesis of Inflammatory Bowel Disease (IBD)

**DOI:** 10.3390/ph14080708

**Published:** 2021-07-22

**Authors:** Alexander S. Dowdell, Sean P. Colgan

**Affiliations:** Department of Medicine and the Mucosal Inflammation Program, Anschutz Medical Campus, University of Colorado School of Medicine, Aurora, CO 80045, USA; alexander.dowdell@cuanschutz.edu

**Keywords:** microbiota, autophagy, mucosa, inflammation, butyrate, indole, GWAS

## Abstract

Inflammatory bowel disease (IBD) is a family of conditions characterized by chronic, relapsing inflammation of the gastrointestinal tract. IBD afflicts over 3 million adults in the United States and shows increasing prevalence in the Westernized world. Current IBD treatments center on modulation of the damaging inflammatory response and carry risks such as immunosuppression, while the development of more effective treatments is hampered by our poor understanding of the molecular mechanisms of IBD pathogenesis. Previous genome-wide association studies (GWAS) have demonstrated that gene variants linked to the cellular response to microorganisms are most strongly associated with an increased risk of IBD. These studies are supported by mechanistic work demonstrating that IBD-associated polymorphisms compromise the intestine’s anti-microbial defense. In this review, we summarize the current knowledge regarding IBD as a disease of defects in host–microbe interactions and discuss potential avenues for targeting this mechanism for future therapeutic development.

## 1. Introduction

Inflammatory bowel disease (IBD) is a family of conditions characterized by chronic, relapsing inflammation of the gastrointestinal (GI) tract [1]. IBD can be sub-divided into two sub-categories of disorders, Crohn’s Disease (CD) and ulcerative colitis (UC), each with dissimilar presentations and pathologies [2]. Although the precise molecular mechanisms of IBD are unclear, it is hypothesized to arise from an aberrant immune response to the intestinal microbiota in genetically susceptible individuals, triggered by as-of-yet unknown environmental stimuli [3]. Epidemiological evidence suggests that the incidence of IBD correlates with industrialization, as IBD was initially described in North America/Europe and has shown increasing prevalence in developing countries/regions, such as South America, China, and Bahrain [4]. However, some evidence exists that disease progression and genetic polymorphisms conferring susceptibility to IBD are heterogenous between different regions and populations, confounding the search for “universal” IBD genes or triggers [5]. Although the majority of risk loci do appear to be shared across individuals of diverse ancestry, some notable differences have been observed including lack of association of European *NOD2* mutations with CD in patients of East Asian ancestry [5,6,7]. Despite the heterogeneity of IBD susceptibility factors, some common themes have arisen through decades of research; for example, high-fat, high-sugar, low-fiber “Western” diets have been demonstrated to increase one’s risk of IBD, possibly through exacerbating IBD-associated intestinal dysbiosis [8]. Likewise, antibiotic therapy resulted in rapid and long-lasting disruption of the gut microbiota, resulting in a pre-disposition to IBD when antibiotic administration occurs early in life [9].

The observation that diet and antibiotic usage influence both IBD susceptibility and the gut microbiota suggests a causative link between IBD and host interactions with intestinal microflora. Indeed, early genome-wide association studies (GWAS) observed an association between innate immune genes and genetic loci associated with IBD, implying that IBD may result, at least in part, from malfunctions in immune responses to microorganisms [10,11]. Further, IBD-associated dysbiosis, or pathologic changes to the composition of the gut microbiota, correlates with the loss of beneficial microbiota-derived metabolites and the presence of colitogenic species [12]. These observations suggest that intestinal homeostasis and the gut microbiota are intrinsically linked, and that loss of homeostasis promotes pathological interactions of the microbiota with the intestinal epithelium. In this review, we discuss the current knowledge regarding the genetic variants that lead to increased susceptibility to pathologic host–microbe interactions, their consequences, and the outlook for future development of therapies that target the dysregulated host–microbe axis in IBD patients.

## 2. Genetic Basis for IBD: Loss of Homeostatic Intestine–Microbe Interactions

IBD patients have been known to have an altered gut microbiota composition relative to healthy individuals since at least 1978, when *Pseudomonas*-like bacteria were found in the tissues of CD patients but not in healthy controls [13]. Since then, numerous studies have demonstrated that the composition of the gut microbiota is altered in IBD patients [14,15,16,17]. These alterations correlate with the loss of beneficial microbiota-derived metabolites, including short-chain fatty acids (SFCAs) and indole derivatives, potentially contributing to IBD pathogenesis [18,19]. Interestingly, one study observed dysbiotic changes in healthy first-degree relatives of IBD patients with accompanying increases in fecal calprotectin (an established biomarker of intestinal inflammation), suggesting that a genetic predisposition towards pathologic host–microbe interactions may be a driving force for disease progression [20,21]. These findings that healthy first-degree relatives of IBD patients may demonstrate genetically driven dysbiosis is reminiscent of other pathologic phenotypes observed in close relatives of IBD patients, including increased intestinal permeability and the detection of serum anti-*Saccharomyces cerevisiae* antibodies [22,23]. These observations, taken together with the longstanding knowledge that children of IBD patients have an increased risk of developing the disorder [24], suggest that, alongside environmental factors, a core driving force for IBD pathogenesis lies in an underlying genetic predisposition.

**Figure 1:**
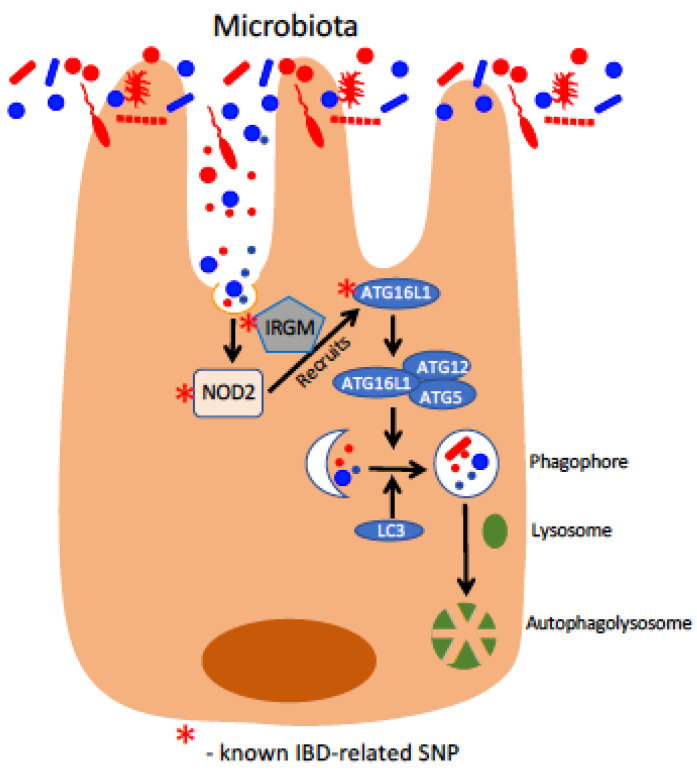
**Defects in epithelial xenophagy responses in IBD.** Shown here is the xenophagic response to luminal microbes and microbial components. These components are recognized by NOD2, which, in conjunction with IRGM, recruits components of the autophagic machinery, including ATG16L1. Upon activation, ATG16L1 associates with the ATG5-ATG12 complex to recruit LC3 in the development of the membrane enclosed phagophore. The cargo-loaded phagophore fuses with cellular lysosomes to form the autophagolysosome in the final degradation and recycling of the xenophagic cargo. Components of this pathway with known IBD-related SNPs are designated with a red asterisk.

The first genetic factor identified to confer susceptibility to IBD was discovered in 2001, when the gene *NOD2*/*CARD15* (hereafter referred to as *NOD2*) was shown to confer increased susceptibility to CD [25,26]. Although a susceptibility locus for IBD had been previously mapped to chromosome 16 [27], studies by Hugot et al. and Ogura et al., published simultaneously in *Nature*, were the first to demonstrate that frame-shift mutations to the NOD2 protein were linked to increased incidence of developing CD. NOD2 is a cytoplasmic pattern recognition receptor (PRR) that recognizes muramyl dipeptide (MDP) [28], a component of peptidoglycan that itself is an element of bacterial cell walls and present in virtually all eubacteria (with the notable exception of the *Mollicutes* class) [29]. When bound to cytoplasmic MDP, NOD2 oligomerizes to activate NF-κB and MAPK signaling cascades (reviewed in detail elsewhere [30]) that culminate with the induction of anti-microbial genes, such as those encoding defensins, and of pro-inflammatory cytokines. The most common identified IBD-associated polymorphisms in NOD2, the amino acid mutations R702W, G908R, and L1007fs, all occur in the C-terminal leucine rich repeat domain responsible for detection of cytoplasmic MDP and have been shown to result in defective sensing of this bacteria-derived molecule [31,32]. NOD2 is expressed in multiple immune and epithelial cell lineages (Figure 1), but shows particularly high expression in cells with a direct role in host–microbe interaction such as ileal Paneth cells and myeloid cells, including dendritic cells (DCs) and macrophages [33]. Correspondingly, NOD2 expression can be induced through bacteria-derived molecules such as lipopolysaccharide and SCFAs, indicative of a central role in the response to bacteria [34,35]. NOD2 plays a crucial role in Paneth cell-mediated regulation of the gut microbiota, and deficiency of functional NOD2 results in compromised secretion of anti-microbial peptides from Paneth cells and overgrowth of commensal microbiota [36,37]. NOD2 is also essential for proper function of other intestinal epithelial cell (IEC) lineages; for example, goblet cell mucin secretion is inhibited in the absence of NOD2, contributing to the pathologic overgrowth of the commensal *Bacteroides vulgatus* [38]. Immune cell function is also dependent on NOD2, with the antigen-presenting ability of DCs and the maintenance of regulatory T cell populations dependent on the activity of this protein [39,40]. Loss of NOD2 has been shown to be detrimental in various murine models of IBD, including the chemically induced dextran sodium sulfate (DSS) and 2,4,6-trinitrobenzenesulfonic acid (TNBS) models and the *Citrobacter rodentium* model of infectious colitis [41,42,43].

Following the discovery of *NOD2* as an IBD susceptibility gene, the search continued for other factors that demonstrated variants with significant association with IBD. In 2007, a GWAS of 735 CD patients identified a single-nucleotide polymorphism (SNP) associated with CD that resulted in a single amino acid substitution (T300A) in the macroautophagy gene *ATG16L1* [44] (Figure 1). Macroautophagy, hereafter referred to as autophagy, is a conserved eukaryotic mechanism for degradation and recycling of cellular components and has been linked to the pathogenesis of a number of disorders including Parkinson’s Disease, skeletal and cardiac myopathies, and osteoporosis [45]. Reviewed in detail elsewhere [46], the process of autophagy is tightly controlled and involves multiple layers of regulation, as uncontrolled autophagy can result in a form of non-apoptotic cell death termed “autosis” [47]. ATG16L1, originally characterized as Apg16p in *S. cerevisiae* by Nobel laureate Yoshinori Ohsumi’s group [48], interacts with the ATG5-ATG12 complex (Figure 1) to mediate lipidation of ATG8 family proteins (LC3s and GABARAPs) and to facilitate their subsequent insertion into the autophagosomal membrane [49]. The discovery by Hampe et al. of ATG16L1 T300A as a CD risk factor was followed later that year by a second study that confirmed this variant’s association with CD and further demonstrated that the loss of ATG16L1 in vitro hampered anti-bacterial autophagy (termed “xenophagy”) of *Salmonella enterica* subsp. *enterica* serovar Typhimurium, a model intracellular bacterium [50]. These two studies were followed by others that demonstrated a role for ATG16L1 in the secretion of anti-microbial factors from Paneth cells and of mucin from goblet cells, as well as showing that the T300A variant conferred increased susceptibility to infection by intracellular bacteria [51,52,53]. The T300A mutation was later found to impart a caspase-3 cleavage site to ATG16L1, resulting in its degradation in response to cellular stress and the subsequent loss of functional ATG16L1, blocking autophagy and clearance of the intracellular bacterium *Yersinia enterocolitica* [54]. Importantly, ATG16L1 was found to directly interact with NOD2 to promote xenophagy in response to detection of cytoplasmic MDP, with this anti-microbial response compromised in epithelial cells bearing CD-associated polymorphisms in either *NOD2* or *ATG16L1* [55]. These results imply that ATG16L1 works with NOD2 to coordinate the response to intracellular bacteria, a hypothesis bolstered by a second study that found ATG16L1 was actively recruited to the plasma membrane by NOD2 at sites of intracellular bacterial entry [56]. As before, CD-associated mutations in *NOD2* were detrimental to the containment of invasive bacteria by xenophagy, as NOD2 variants were deficient in the recruitment of ATG16L1 to the plasma membrane. Further studies found that ATG16L1 has an important regulatory role in modulating the pro-inflammatory functions of activated NOD2 and that, in the context of CD-associated ATG16L1 variants, stimulation of NOD2 with bacteria-derived ligands promotes a NOD2-mediated pathologic inflammatory response [57,58]. Interestingly, the property of ATG16L1 to coordinate an anti-microbial response with NOD2 has been found in some circumstances to be independent of autophagy, suggesting a novel role for ATG16L1 distinct from its initially described purpose as part of the ATG5-ATG12-ATG16L1 E3 complex [58] (Figure 1). The “multi-functional” nature of ATG16L1 is in line with the accumulating evidence that many ATG proteins “moonlight” in non-autophagy roles, reviewed in detail elsewhere [59]. Furthermore, the importance of functional NOD2 and ATG16L1 in maintaining gut homeostasis extend to beyond the epithelium. Both proteins were found to be essential for proper antigen presentation and bacterial handling in dendritic cells, as well as the maintenance and activity of regulatory T cells [39,40,60].

In 2007, a GWAS of CD patients and healthy controls by Parkes et al. identified variants the gene *IRGM* as strongly associated with CD [61]. IRGM, the sole human homolog of the immunity-related GTPase (IRG) family, had been previously demonstrated to be regulated by interferon-γ and important for control of intracellular bacteria, especially *Mycobacterium tuberculosis*, through induction of autophagy [62,63] (Figure 1). Similarly, mice lacking the murine IRGM homolog LRG-47 were highly susceptible to the intracellular pathogens *Toxoplasma gondii* and *Listeria monocytogenes* [64]. Although Parkes et al. did not determine the mechanism by which the IRGM variants imparted susceptibility to CD, subsequent studies found that alterations in IRGM expression levels, rather than coding mutations to IRGM itself, resulted in compromised xenophagy and increased risk of CD [65,66]. Further studies found that IRGM interacts with several other known autophagy proteins, including MAP1LC3C and ATG5, and targets the NLRP3 inflammasome for selective autophagy to prevent pathologic gut inflammation in vivo [67,68]. Notably, IRGM was also found to associate with NOD2 and ATG16L1 to orchestrate the xenophagic response to intracellular bacteria (Figure 1), resulting in association with other autophagy proteins, clearance of intracellular bacteria, and suppression of pro-inflammatory responses [69]. IRGM has also been shown, interestingly, to display splice isoform-dependent regulation of mitochondria, with specific isoforms promoting mitochondrial depolarization and cell death [70]. These findings suggest that IRGM may influence cellular homeostasis through mechanisms distinct from those involved in the response to intracellular microbes. Finally, IRGM and ATG16L1 have both been shown to be important for the xenophagic control of intracellular adherent-invasive *Escherichia coli* (AIEC), a strain distinctly associated with IBD dysbiosis [71,72].

Aside from *NOD2*, *ATG16L1*, and *IRGM*, other genes have been implicated in the pathogenesis of IBD. XIAP (X-linked inhibitor of apoptosis) is a E3 ubiquitin ligase that ubiquitinates the serine/threonine/tyrosine protein kinase RIPK2 (Receptor Interacting Serine/Threonine Kinase 2) upon RIPK2′s activation by MDP-bound NOD1/2 [73]. RIPK2 is a crucial adaptor protein in the transduction of NOD1/2 signaling and is essential for proper downstream activation of NF-κB [74]. Further, RIPK2 is necessary for the cellular response to numerous microorganisms, and loss of RIPK2 has been demonstrated to compromise xenophagy [75,76]. Although no IBD-associated polymorphisms in *RIPK2* have been identified as of yet, mutations in its activator *XIAP* have been linked to very early onset (VEO)-IBD in males through loss of XIAP ubiquitinating activity and subsequent inhibition of NOD/RIPK2 signaling [77,78,79]. These findings highlight the importance of the NOD2 signaling cascade in maintenance of intestinal homeostasis, and suggest that polymorphisms that interrupt the sensing of bacterial products (e.g., MDP) may drive intestinal inflammation. Besides *XIAP*, polymorphisms in other genes modulating such biological functions as intestinal barrier integrity (*HNF4A*, *PTGER4*), adaptive immunity (*IL12B*, *STAT4*), and inflammasome regulation (*MEFV*) have all been implicated in susceptibility to various forms of IBD, suggesting that the driving forces for IBD are complex and unique to each patient [80,81,82,83,84,85].

## 3. Role of Microbiota in Modulating Intestinal Barrier and Inflammation

A recent meta-analysis of GWAS data from UC and CD patients notes a significant overlap in susceptibility loci between IBD and mycobacterial infection, implying that a response to intracellular bacteria could underlie the pathology of IBD [86]. This is in agreement with clinical observations that antibiotic therapy can improve IBD symptoms under some circumstances, presumably through a reduction in gut bacterial load and/or modulation of microbiota composition [87]. The gut microbiota, composed of over 10^14^ microorganisms (eubacteria, archaea, fungi, etc.) and >1000 bacterial species, has been demonstrated to be essential for the spontaneous development of colitis in the Il-10^−/−^ mouse model of IBD [88,89]. Similarly, induction of intestinal inflammation through adoptive transfer of CD4^+^CD45RB^hi^ T cells is dependent on the presence of the intestinal microbiota, as inflammation is not seen in animals devoid of most intestinal bacterial species [90]. Increased serum concentrations of bacterial lipopolysaccharide (LPS) can be measured in patients with active IBD compared to healthy controls, suggesting a breakdown in intestinal epithelial barrier function and/or increased translocation of bacterial across the gut epithelium [91,92]. Interestingly, patients with inactive CD were still found to have elevated serum LPS, suggesting a baseline defect in intestinal homeostasis that fails to resolve even with the remission of overt CD symptoms [91]. These conclusions were reflected in a separate study that found bacterial DNA to be detectable only in IBD patients’ serum, regardless of active/inactive disease status, suggesting that enhanced intestinal permeability to bacteria and their products is an underlying feature of IBD [93].

The precise role of the microbiota in gut homeostasis and in the pathogenesis of GI inflammatory disorders is complex and highly dependent on many factors, including the individual’s genetic background, the microbial species present, and poorly characterized environmental determinants [94]. Within a single individual, the gut microbiota can vary dramatically due to factors such as age, diet, exercise frequency, and antibiotic usage [95]. Such confounding factors complicate efforts to discern a “healthy” microbiota from a “pathologic” microbiome; nevertheless, sequencing of the microbiome in IBD patients has revealed common threads that demonstrate the malleability of the microbiota through the course of disease onset and relapse [96]. A recent systematic review found that certain enteric pathogens (such as *Salmonella* and *Norovirus* spp.) were positively correlated with IBD flares, possibly through pathologic inflammatory stimulation in dysregulated microbe-sensing pathways (e.g., NOD2), whereas others (including *Helicobacter pylori* and various helminths) were protective in the context of IBD [97]. Interestingly, *H. pylori* seems to demonstrate a negative correlation with IBD, possibly through induction of immunogenic tolerance of gut microbiota; however, the demonstrated association of *H. pylori* and gastric cancer suggests that the molecular mechanisms by which *H. pylori* regulates intestinal inflammation may be of more interest clinically than the bacterium itself [97,98,99]. Analyses of IBD/non-IBD gut microbiomes reveal consistent disease-associated patterns, including decreased α-diversity, increased abundance of facultative anaerobes accompanied by loss of obligate anaerobes, and pathologic alterations in microbiota-derived metabolites [18,19,100,101]. The latter is especially noteworthy, as addressing imbalances in gut metabolites through small molecule supplementation is a highly accessible strategy for treatment of human disease [102].

**Figure 2:**
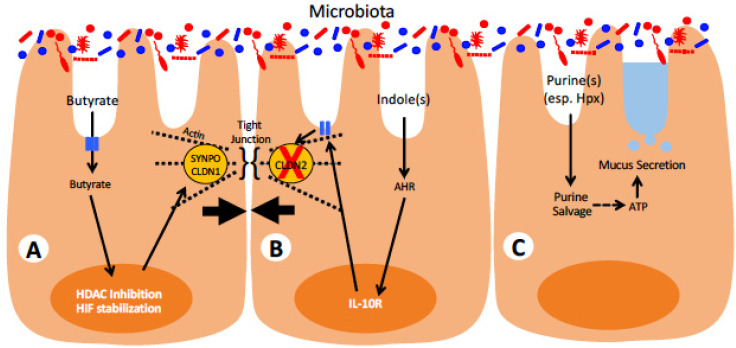
**Microbial-derived metabolites and known functions on intestinal epithelial barrier function.** Cell **A** depicts known response to the short-chain fatty acid butyrate. Once taken through apical membrane transporters, butyrate function as both an HDAC inhibitor and HIF stabilizer to promote expression of tight junction-associated proteins, including synaptopodin (SYNPO) and claudin-1 (CLDN1), resulting in enhanced epithelial barrier function. Cell **B** shows epithelial responses to the microbial tryptophan derivative indole. Once inside cells, indole(s) associate with the arylhydrocarbon receptor (AHR) to activate transcriptional induction of the interleukin-10 receptor (IL-10R), which upon activation, results in the loss of “leaky” claudin-2 (CLDN2), thereby promoting epithelial barrier function. Cell **C** represents the most recent observations that various microbial-derived purines (esp. hypoxanthine, Hpx) are recycled via purine salvage to be used as an energy source. Increases in intracellular ATP are associated with enhanced mucus secretion to promote enhanced epithelial barrier function.

Recent findings by our group and others have demonstrated that microbiota-derived metabolites are essential for maintaining intestinal barrier homeostasis, and loss of these metabolites through the course of intestinal inflammation may be a driving force for IBD pathogenesis (Figure 2). Through fermentation of undigestible fibers, select members of the gut microbiota generate SCFAs, particularly acetate, propionate, and butyrate, that have been found to exert influences on diverse biological processes [103]. Butyrate especially been shown to potentiate intestinal epithelial barrier function through modulation of actin-binding proteins and by serving as the primary fuel source for colonocytes [104,105,106]. When intestinal butyrate concentrations are insufficient, as observed in germ-free mice or during acute intestinal inflammation, colonocytes become energy deficient and are unable to maintain normal levels of ATP and the reducing agent NADH [107]. Original studies by Roediger suggested that oxidation of butyrate by colonocytes from ulcerative colitis patients was defective and resulted in an energy-deficient (“starved”) mucosa [108]. As maintenance of intestinal barrier function is an energetically taxing process, loss of cellular energy homeostasis compromises the ability of IECs to maintain intestinal barrier function and results in increased translocation of bacteria into the lamina propria. Butyrate also serves to maintain normal intestinal epithelial function in other ways including downregulation of the “leaky” tight junction protein Claudin-2, induction of “tight” claudin Claudin-1 and stabilization of the transcription factor HIF-1α (Figure 2), the latter of which has been repeatedly shown to be protective in murine models of colitis [109,110,111,112,113,114]. Butyrate and other SCFAs also have influences on immune functions, particularly through their ability to induce protective regulatory T cell responses in the gut [115,116]. The role of SCFAs in regulating immune functions is of particular importance in IBD given its hypothesized etiology and are reviewed in detail elsewhere [117]. Notably, butyrate has been demonstrated to directly shape the composition of the gut microbiota, either through suppression of virulence genes in pathogenic bacteria or by increasing epithelial oxygen consumption and preventing subsequent outgrowth of facultative anaerobes [118,119]. The loss of SCFA-producing obligate anaerobes, as occurs during antibiotic treatment, can therefore cause runaway intestinal inflammation through gut oxygenation and intestinal dysbiosis, preventing re-establishment of SCFA producers and normalization of gut homeostasis [120]. SCFAs are also potent histone deacetylase (HDAC) inhibitors and have been shown to exert epithelial protective effects through inhibition of IEC HDACs, in addition to their role in maintaining cellular energy homeostasis [104,114,121]. Import of SCFAs into IECs is accomplished through several fatty acid transporters, including SLC16A1 (MCT1), SLC16A3 (MCT4), SLC5A8 (SMCT1), and SLC5A12 (SMCT2) as reviewed elsewhere [122]. SCFAs can also modulate cellular activity without intracellular transport through binding to a number of G protein-coupled receptors (GPCRs), such as FFAR2 (GPR43), FFAR3 (GPR41), HCAR2 (GPR109A), and OR51E2 (OLFR78) [123]. As reviewed in detail elsewhere, binding of extracellular SCFAs to SCFA-sensing GPCRs in both epithelial and immune cells triggers numerous signaling cascades that result in modulation of diverse cellular functions including regulation of inflammatory state, secretion of endocrine factors, and alteration of immune cell chemotaxis [123,124,125]. The responses of the GPCRs seem to be dependent on a number of factors, including the particular SCFA agonist, the expressing cell type, the host species, and, in some cases, the bound G_α_ subunit. Nevertheless, the potential accessibility of SCFA-activated GPCRs to pharmacological intervention has made them prospective targets for development of novel IBD therapeutics [126].

In addition to SCFAs, the gut microbiota is also a rich source of purines that have been found to be essential for gut epithelial homeostasis. In particular, microbiota-derived hypoxanthine has been demonstrated to be beneficial for epithelial barrier function by serving as an ATP precursor, increasing the pool of easily available energy to the cell much and protecting against energetically stressful events, such as acute inflammation, much in the same way as creatine [127,128,129]. Importantly, colonic hypoxanthine concentrations were found to inversely correlate with disease metrics in colitic mice, suggesting that loss of this microbial-derived purine accelerates disease progression [127]. To this end, supplementation of streptomycin-treated mice with exogenous hypoxanthine is protective in the DSS model of acute colitis [130]. As streptomycin-treated mice show loss of extracellular purines in intestinal contents, these results suggest that restoration of intestinal purines is protective through increasing epithelial homeostasis, as evidenced by increased mucosal layer thickness and Ki-67 staining. The observation that microbiota-derived metabolites (SCFAs and purines) are involved with cellular energetics suggests that maintenance of intestinal homeostasis is highly dependent on a symbiotic relationship with commensal gut microbiota for cellular metabolism [131] (Figure 2).

Lastly, gut metabolism of the essential amino acid tryptophan has attracted significant attention for its role in mediating intestinal homeostasis and for its dysregulation during intestinal inflammation, with subsequent extra-intestinal consequences [132]. Tryptophan metabolites play diverse roles in both host–microbe and microbe–microbe interactions, with the latter possibly affecting the composition of gut microbial communities at a cross-kingdom scale [133]. With regard to host–microbe interactions, tryptophan metabolites typically activate the aryl hydrocarbon receptor (AHR) pathway, akin to dioxins and other aromatic xenobiotic hydrocarbons, resulting in a complex transcriptional response that is generally beneficial and prevents pathologic immune activation and interactions with gut microbiota [134] (Figure 2). During episodes of acute intestinal inflammation, microbial tryptophan metabolism can become disrupted due to inflammation-induced dysbiosis, resulting in alterations to serum and colon levels of tryptophan metabolites including kynurenine and various indole derivatives [135,136]. Supplementation of mice during DSS colitis with these compounds revealed that tryptophan metabolites are protective in the context of acute intestinal inflammation, at least in part through AHR-dependent induction of IL-10R1 on epithelial cells. Subsequent studies using bacteria-derived indole compounds found that they specifically inhibit neutrophil myeloperoxidase, a key inflammatory mediator during acute colitis, and suggest that microbial-derived tryptophan metabolites can directly act on innate immune cells to attenuate the inflammatory response [137].

## 4. Approaches to Intervene in Modulating Host–Microbe Interactions

Current IBD treatment regimens follow schemes designed to initially resolve intestinal inflammation, then prevent recurrence of subsequent inflammatory bout (“flares”) through management of immune activity with specific recommendations for UC and CD [138]. Given the central role of the gut microbiota in mediating intestinal homeostasis (see above), strategies to mold the gut microbiota in ways that may benefit IBD patients have been proposed and attempted, with some success [139]. This is an area of intense investigation. For a summary of the following section, please refer to Table 1.

The best studied intervention that directly acts upon the microbiota is fecal microbiota transplantation (FMT). In FMT, fecal material from a healthy donor is transferred directly to a patient through endoscopy, enema, or capsules ingested orally, with the ultimate goal of reversing microbiota dysbiosis [140]. FMT has been conclusively demonstrated to improve outcomes in refractory *Clostridium difficile* infections. While FMT is thought to “reset” the microbiota, the actual mechanisms are unclear. For example, in a small cohort of *C. difficile* infected patients it was recently shown that sterile filtered fecal samples were as effective as non-filtered samples in the resolution of disease [141]. FMT has attracted attention as a potential therapy for IBD given the proposed role of the microbiota in the latter’s etiology [142,143]. One recent case report detailed the induction of remission in a patient with steroid-refractory UC using FMT [144]. This case was notable in that the patient displayed an allergy to 5-ASA, a first-line therapy for inducing and maintaining UC remission in mild to moderate cases [145], and therefore represents a potential alternate strategy for patients for whom 5-ASA (and other drugs) is not tolerated or has lost effectiveness. Similarly, FMT was shown to improve endoscopic/histologic remission in UC patients and prevent relapse, suggesting a role for FMT in maintaining intestinal homeostasis in inflammation-prone patients [146]. However, a recent Cochrane review found that the overall evidence for the use of FMT in UC was weak given the studies published at the time (2018) and suggested that more clinical trials commence before any recommendation could be given. The review also found no quality studies that addressed the efficacy of FMT in the context of CD, indicating a particular need for controlled trials in CD patients. Other systematic reviews have indicated a potential benefit for FMT in treating CD, and one recent controlled trial demonstrated significant positive effects of FMT in the decrease in the endoscopic index of severity and prevention of C reactive protein elevation (a serum marker for inflammation) in CD patients versus sham-treated controls [147,148,149]. One explanation for the potential variability of results observed in FMT clinical trials is the exquisite sensitivity of “beneficial” gut bacteria to oxygen: oxygen toxicity, as well as fastidious nutritional requirements and other unknown factors, has been a major hurdle in the culturing of novel bacterial species from fecal matter [150]. Oxygen exposure has also been shown to diminish bacterial diversity of donor stool and resulted in significant loss of *Faecalibacterium prausnitzii*, an SCFA producer with documented positive roles in intestinal homeostasis, immune regulation, and amelioration of disease in animal models of colitis [151,152]. The hypothesis that handling anaerobic handling/preparation of donor stool for FMT preserved “beneficial” species is supported by a recent clinical study that achieved steroid-free remission in nearly 1/3 of treated UC patients using anaerobically prepared samples, suggesting a positive benefit in the exclusion of oxygen from the sample preparation process [153]. Further studies should focus on the efficacy of FMT in different types of IBD, especially CD, with particular attention paid towards preservation of sensitive microbial species in the donor stool.

The use of specific species of “beneficial” bacteria has attracted attention for similar reasons as FMT, in that positive aspects of gut microbiota composition could influence disease outcomes through immune modulation and restoration of homeostatic host–microbe interactions [154]. The bacteria *Bifidobacterium longum* 536 and *Escherichia coli* Nissle 1917, for example, have been shown to improve outcomes in UC patients, with the latter observing *E. coli* Nissle 1917 treatment to be as effective as mesalazine (5-ASA) in maintaining disease remission [155,156]. Probiotic bacteria have been described to exert protective effects through potentiation of intestinal concentrations of SCFAs, either through direct production or by promoting the growth of other SCFA-generating species [157,158]. Probiotics also mediate resistance to pathogenic bacteria through outcompeting pathogens for vital nutrients, such as iron [159]. Another study found that a cohort of proteins from the probiotic *Propionibacterium freudenreichii* act synergistically to induce IL-10 expression in vitro, suggesting that probiotic bacteria may have evolved to regulate host inflammation through tailoring of their proteomes [160]. One probiotic, *Lactococcus lactis*, has been successfully engineered to secrete bioactive IL-10, with the resulting strain observed to be protective in both the DSS and IL-10^−/−^ models of murine colitis [161]. Human clinical trials using IL-10-secreting *L. lactis* have been conducted, with the result that the bacterium was well tolerated and biologically contained due to a *thyA* auxotrophic mutation; however, outcomes were mixed with some protection observed in CD patients but none observed in a separate study with UC patients [162,163]. These disparate results indicate that optimizations to the IL-10 expression strain and/or to the bacterial delivery method may be warranted for clinical trial results to recapitulate the successes observed in animal studies. Further, recent studies have investigated the role of non-bacterial probiotics in the treatment of intestinal inflammatory disorders. Oral administration of the yeast *Saccharomyces boulardii* has been demonstrated to reduce disease metrics in the CD4^+^CD45RB^hi^ T cell adoptive transfer, DSS, and *C. rodentium* models of intestinal inflammation, suggesting a protective role for this organism in attenuating inflammation [164,165,166,167]. Administration of *S. boulardii* has also been observed to improve intestinal permeability defects in CD patients when combined with existing therapies [168]. Although the mechanisms by which *S. boulardii* exerts a protective influence in the intestine are poorly understood, it is hypothesized that *S. boulardii* is protective through regulation of miRNA expression, inhibition of NF-κB, and modulation of the gut microbiota [167,169,170]. At least one clinical study (NCT03941418) has been organized to investigate the potential therapeutic role of *S. boulardii* in treatment of IBD. Recently, a strain of *S. cerevisiae* has been engineered to sense extracellular ATP (eATP) in the gut and respond by secretion of recombinant apyrase, catalyzing the degradation of eATP to eAMP [171]. The resulting eAMP is further degraded to extracellular adenosine by CD73, which is ubiquitously expressed on the apical face of the intestinal epithelium; the resulting extracellular adenosine (eAdo) has been shown to be protective during intestinal inflammation through diverse mechanisms [172,173,174]. Scott et al. demonstrate that eAdo-generating *S. cerevisiae* attenuates intestinal inflammation during chemically induced colitis models (DSS and TNBS) and show that engineered *S. cerevisiae* administration limits inflammation induced fibrosis and dysbiosis [171]. These results further highlight the importance of nucleotide/nucleoside signaling in the gut and suggest that intervention with engineered yeast is a feasible approach for future generation of novel therapeutics. Despite their promising preliminary results, the use of probiotics in the treatment of intestinal inflammation is not without caveats. At least one case report has described an instance of bacteremia in an adult patient with severe active UC due to self-administered probiotics, indicating that the usage of probiotics in the context of IBD is likely best undertaken under the supervision of medical professionals [175]. Additionally, two Cochrane reports found no evidence to support the use of probiotics for the induction or maintenance of remission in CD, indicating that further studies are warranted before any recommendations could be made regarding probiotic use in this disorder [176,177].At least three clinical trials (NCT00175292, NCT01078935, NCT01772615) have also been established to evaluate the potential therapeutic potential of probiotics in the amelioration of IBD. Taken together, these results suggest that the use of probiotic microbes may hold promise for treatment of IBD, but further research is needed before firm conclusions can be drawn.

Administration of bacterial metabolites themselves has been investigated as a potential means by which IBD can be treated in a manner consistent with in vivo regulation of intestinal homeostasis [12]. As previously discussed, IBD patients display distinct metabolite patterns that reflect, in part, the microbial dysbiosis that accompanies intestinal inflammation, with particular metabolites offering promising avenues for intervention [178]. One such metabolite is butyrate, a microbial-derived metabolite demonstrated to be protective in animal models of IBD [179]. Evidence from clinical trials suggests that butyrate enemas are safe and efficacious in improving resolution of distal ulcerative colitis and, when combined with the first-line agent 5-ASA, results in a greater improvement in symptoms than use of 5-ASA alone [180,181,182]. Although a recent systematic review found that the use of butyrate enemas in treatment of UC was unsupported by existing results, the review’s authors noted the limited availability of data concerning butyrate use in UC and the absence of reliable data in CD patients [183]. Although no clinical trials concerning SCFA application in IBD have resulted in a therapeutic entering the market, the importance of SCFAs to intestinal health cannot be understated and remains a tempting avenue for future research. In addition to SCFAs, microbiota-derived indole derivatives show promise as potential therapeutics for IBD. In mouse models of IBD, the metabolite indole-3-propionic acid (IPA) dramatically alleviated DSS-mediated intestinal inflammation resulting in restored intestinal tissue architecture and a reduction in inflammatory parameters, such as histologic score and colon shortening [136]. IPA, exclusively produced from tryptophan by distinct subsets of the gut microbiota, has been implicated in both regulation of innate immune activity and suppression of gut inflammation through synergistic activation of the pregnane X receptor with another microbiota-derived tryptophan metabolite, 1*H*-indole [137,184,185,186,187]. IPA also demonstrates potent anti-oxidant activity and shows neuroprotective characteristics in models of neurodegenerative disorders such as Alzheimer’s disease and ischemic stroke [188,189]. As a neuroprotectant, IPA is currently undergoing development for treatment of Friedrich’s ataxia, a neurodegenerative disorder; a recent clinical trial (NCT01898884) found that oral administration of IPA was well tolerated with only mild side-effects, suggesting that its potential future evaluation for treatment of IBD would be reasonable. Given the notable reduction in gut inflammation observed in animal models and its in vivo safety profile, IPA holds potential as a promising future IBD therapeutic. A recent randomized control trial also found that administration of indigo naturalis (which is enriched in indole-like molecules) to patients with active UC improved disease metrics such as clinical remission and mucosal healing, presumably through induction of IL-22 via the AHR pathway [190]. It is entirely possible that these microbiota-derived metabolites may exert synergistic effects in vivo, given their co-existence in the “healthy” gut—such synergism may account for the differences observed between in vitro experiments, animal studies, and clinical trials. Future studies should seek to investigate whether the protection afforded by such molecules as butyrate and IPA is amplified when administered in tandem, rather than as individual interventions.

Lastly, bacteriophages (“phages”) have been speculated as a potential future therapy to treat IBD through modulation of gut bacterial composition [191]. Phages are a ubiquitous, though often overlooked, component of the gut microbiota, with one study finding over 1000 viral species in a single adult by metagenomic sequencing [192]. Phages exist in a complex relationship with their host gut bacteria and significant diversity exists between individuals [193]. It has been observed, however, that acute intestinal inflammation causes a dysbiotic alteration in phage populations, similar to that seen in bacterial and fungal communities, with decrease in overall phage diversity accompanied by expansion of specific phage subsets [194]. These observations are reminiscent of the similar dysbiotic shifts seen in gut eubacterial populations and suggests that the two populations (phage and eubacteria) are inexorably linked. In a separate study, researchers found that phages could directly stimulate the immune system, possibly through transcytosis across the intestinal epithelium, and that stimulation of the immune system by phages can potentiate colitis through TLR9-mediated interferon-γ production [195]. Despite this role in intestinal inflammation, phages have been shown to be beneficial to target specific, pathogenic members of the gut microbiota, a strategy termed “phage therapy” [196]. Phages targeting the IBD-associated pathobiont AIEC show efficacy in reducing bacterial colonization, bacteria-exacerbated intestinal inflammation, and the development of intestinal tumors in the APC^min^ mouse model [195,197]. Further, *C. difficile* was successfully treated using phage therapy [198], including one ingenious study that utilized a phage-delivered CRISPR-Cas3 system to directly target the bacterial chromosome [199]. Given the rising incidence of antibiotic resistance [200] and the tendency of antibiotics to indiscriminately eliminate gut bacteria, including beneficial symbionts, phage therapy offers a novel, highly targetable approach to modulating the intestinal microbiota that, while still highly experimental, holds promise for future development as an IBD therapy. Multiple clinical trials (NCT04737876, NCT03808103) have recently investigated the safety and tolerability of phage therapy in IBD patients, indicating that future IBD therapeutics treatment regimens may include phage therapy as a direct means of modulating the gut microbiota.

## 5. Conclusions

The gastrointestinal tract plays host to trillions of microbes, collectively termed the microbiota. Recent evidence strongly implicates shifts in the microbiota in IBD. It remains unclear to what extent host factors and microbial factors contribute to IBD disease pathogenesis. In this review, we have evaluated the current scientific knowledge as it relates to regulation of the intestinal mucosa through signaling via endogenous factors. In vitro and in vivo studies, including ones utilizing human IBD tissue, have provided novel insight into the role of host–microbe factors in disease progression and resolution. These studies have revealed a critical role for host handling of microbial components as outcomes for productive innate immunity. Ongoing work will seek to compare and contrast the innate and adaptive immune responses to such stimuli, as well as their role in acute and chronic intestinal inflammation. Further studies will likely provide new insight into disease mechanisms, informing the development of novel IBD therapies.

## Figures and Tables

**Table 1:** List of intestine-protective interventions described in this review.

Treatment	Model	Outcome	Reference
Fecal microbiota transplantation (FMT)	Case report, patient with steroid-refractory UC	Induction/maintenance of remission	[144]
	Randomized controlled trial, UC patients	Maintenance of steroid-free remission	[146]
	Randomized controlled trial, CD patients	Decrease in the endoscopic index of severity, prevention of increase in serum CRP	[149]
*Faecalibacterium prausnitzii*	TNBS mouse model of colitis	Attenuation of colitis, reduction in colitis-driven gut dysbiosis	[152]
*Bifidobacterium longum* 536	Randomized controlled trial, UC patients	Significant decreases in the disease activity index, the Rachmilewitz endoscopic index, and the Mayo subscore	[155]
*Escherichia coli* Nissle 1917	Clinical trial, UC patients	Equivalent to mesalazine in preventing relapse	[156]
IL-10-secreting *Lactococcus lactis*	DSS, Il-10^−/−^ mouse models of colitis	Reduction in intestinal histopathology	[162]
	Clinical trial, CD patients	Decrease in disease activity, serum CRP	[164]
*Saccharomyces boulardii*	Randomized controlled trial, CD patients	Decrease in intestinal permeability	[157]
	*C. rodentium* mouse model of colitis	Decreases in body weight loss, histopathology, tissue MPO, and intestinal permeability observed	[165]
	CD4^+^CD45RB^hi^ T cell adoptive transfer mouse model of colitis	Decreases in body weight loss, histopathology, intestinal pro-inflammatory cytokines, and NF-κB activation observed	[166]
	DSS mouse model of colitis	Reduction in clinical score, histopathology, and colonization by colitis-associated *Candida albicans*	[167]
	DSS mouse model of colitis	Reduction in the disease activity index, improved weight recovery, amelioration of colitis-driven gut dysbiosis	[168]
Extracellular ATP-degrading *S. cerevisiae*	TNBS, DSS, and anti-CD3 mouse models of intestinal inflammation	Decreases in colon length shortening, histopathology, weight loss, and intestinal pro-inflammatory cytokine expression	[171]
Hypoxanthine	DSS mouse model of colitis; mice pre-treated with streptomycin	Decreases in ER stress, intestinal epithelial apoptosis, body weight loss, and colon shortening; increases in mucus secretion, energy homeostasis, and cellular proliferation	[130]
Indole-3-propionic acid (IPA)	DSS mouse model of colitis	Decreases in histopathology, intestinal pro-inflammatory cytokines, and colon shortening	[136]
	Indomethacin mouse model of intestinal inflammation	Reduction in intestinal permeability	[187]
Indigo naturalis	Randomized controlled trial, active UC patients	Increases in clinical remission and mucosal healing	[190]
Butyrate	DSS mouse model of colitis	Decreases in histopathology, colon shortening, pro-inflammatory cytokines in colon tissue	[179]
	Clinical trial, treatment-refractory UC patients	Decreases in endoscopic score, histologic degree of inflammation, stool frequency, and blood discharge	[180]
	Clinical trial, treatment-refractory UC patients	60% response rate based on positive change in activity score	[181]
	Clinical trial, UC patients	Significantly greater improvement in the disease activity index when butyrate combined with 5-ASA vs. 5-ASA alone	[182]
Phage therapy	DSS mouse model of colitis; mice pre-colonized with AIEC	Anti-AIEC bacteriophages reduced the disease activity index and gut AIEC burdens	[197]
	*C. difficile* Syrian Golden hamster model of colitis	Reduced bacterial colonization and delay in symptom onset	[198]
	Cefoperazone-pretreatment/*C. difficile* mouse model of colitis	Reduction in intestinal *C. difficile* burdens	[199]

## Data Availability

Data sharing not applicable.
